# Word Embedding Distribution Propagation Graph Network for Few-Shot Learning

**DOI:** 10.3390/s22072648

**Published:** 2022-03-30

**Authors:** Chaoran Zhu, Ling Wang, Cheng Han

**Affiliations:** College of Computer Science and Technology, Changchun University of Science and Technology, Changchun 130022, China; z2653888596@gmail.com (C.Z.); hancheng@cust.edu.cn (C.H.)

**Keywords:** few-shot learning, graph neural network, semantic information, attention mechanism, Mahalanobis distance, FReLU

## Abstract

Few-shot learning (FSL) is of great significance to the field of machine learning. The ability to learn and generalize using a small number of samples is an obvious distinction between artificial intelligence and humans. In the FSL domain, most graph neural networks (GNNs) focus on transferring labeled sample information to an unlabeled query sample, ignoring the important role of semantic information during the classification process. Our proposed method embeds semantic information of classes into a GNN, creating a word embedding distribution propagation graph network (WPGN) for FSL. We merge the attention mechanism with our backbone network, use the Mahalanobis distance to calculate the similarity of classes, select the Funnel ReLU (FReLU) function as the activation function of the Transform layer, and update the point graph and word embedding distribution graph. In extensive experiments on FSL benchmarks, compared with the baseline model, the accuracy of the WPGN on the 5-way-1/2/5 shot tasks increased by 9.03, 4.56, and 4.15%, respectively.

## 1. Introduction

Most successful deep-learning architectures are based on rich labeled datasets. However, in special practical application scenarios, only small numbers of labeled data may be available, owing to certain limitations. Therefore, there is a need to acquire information on new classes based on limited labeled data. This is known as few-shot learning, in which tasks use a small number of labeled samples to predict unlabeled ones. There are a variety of approaches that have been proposed for FSL to address the deficiency of labeled data.

Meta-learning is one of the main methods used in FSL. Model-Agnostic Meta-Learning (MAML) [1] uses an initialization parameter that requires only a few samples to perform gradient descent and to achieve good results when encountering new problems. Because the MAML method only adjusts parameters according to different tasks, the trained model is prone to overfitting. Task Agnostic Meta-learning for few-shot Learning (TAML) [2] is an improvement on the MAML algorithm. The original design explicitly requires that the parameters of the model have no preference for different tasks during regularization. Meta-learning with memory Augmented Neural Networks (MANN) [3] uses a recurrent neural network (RNN) to memorize the representation of the previous task. Although this method is helpful for learning new tasks, the weight of the RNN updating process is still slow, which makes the training process difficult. Meta-learning with differentiable closed-form solvers [4] uses simpler differentiable regression methods that have closed-form solutions to replace the original learning algorithms (e.g., the *k*-nearest-neighbor (KNN) algorithm and the convolutional neural network) and has inspired our idea of combining a traditional learning algorithm with a neural network. Belonging Network (BeNet) [5] uses basic statistical information of the target class to find the simple mean and variance information to improve performance of the training image set. Regularization using knowledge distillation when learning small datasets [6] leverages the knowledge distillation method, which found that increasing the distillation parameter “temperature” can improve model accuracy, especially for small numbers of training data. However, if the testing and training sets show a large difference with regard to their distribution, the output of the model will be highly unsatisfactory. Task-Aware Feature Embeddings for low-shot learning (TAFE-Net) [7] innovatively uses the meta-learning method to dynamically select weight parameters. According to various tasks, different weight parameters are selected, and the weight decomposition method is used to render this computationally possible. Because the few-shot dataset has no corresponding class description information, the meta-learner’s ability to represent the embedded features of the task is affected. Hence, the experimental effect is only slightly improved compared with other algorithms. Therefore, how to effectively use the limited dataset information has become the focus of our research.

Metric learning maps images into an embedding space where images of the same class are located close to each other, and the images of different classes are farther apart. The Siamese Neural Network [8] limits the structure of the input images and can automatically discover the generalizable features of the new samples. However, because it is sensitive to the differences between the two images, it may easily result in misclassification. Matching networks [9] construct an end-to-end nearest-neighbor classifier. Through meta-learning training, the classifier can quickly adapt to new tasks with few samples. However, when the label distribution has obvious deviations (e.g., being fine-grained), the model becomes unusable. Few-shot image classification with Differentiable Earth Mover’s Distance and structured classifiers (DeepEMD) [10] splits an image into multiple tiles and introduces a new distance measurement method (i.e., Earth Mover’s Distance (EMD)) and calculates the best matching cost between each tile of the query set and the support-set image, which indicates the degree of similarity between the two. Boosting few-shot learning with adaptive margin loss [11] improves the classification effect of the original algorithm by introducing adaptive margin loss of class-relevant or task-relevant information and uses the semantic similarity between different categories to generate adaptive edges. However, there is no correlation between the two proposed edge generation methods. Improved few-shot visual classification [12] uses the Mahalanobis distance to calculate the distance between samples; however, it focuses largely on dividing the most accurate inter-class interval for the existing samples and neglects learning the image features. The metric learning above makes us realize the importance of a distance measurement in FSL.

**Figure 1:**
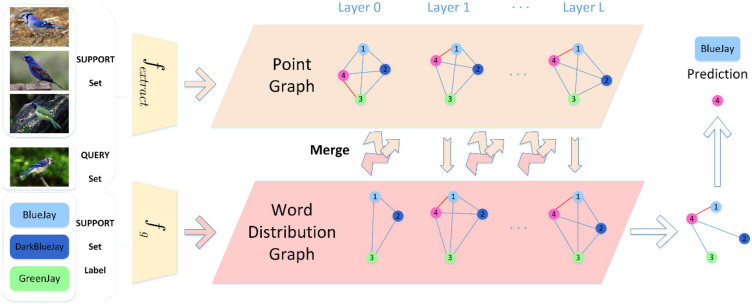
The figure shows a 3-way-1 shot task, where 1, 2, 3 represent the support set, 4 represents the query set, and the red line segment represents the mini-mum similarity between 4 and other support set samples. After cyclic calculation of the L-layer Point Graph and Word Distribution Graph, the class output with the closest distance between 4 and the other three types of samples in the Word Distribution Graph is finally selected as the prediction result.

In this study, we propose a new model for FSL called the word embedding distribution propagation graph network. As illustrated in Figure 1, the word embedding distribution graph is merged into the GNN, and the fine-grained few-shot classification task is solved by the cyclic calculation method. The contributions of this work are summarized below.

First, the WPGN uses the GloVe model to extract the class label information as a word vector. The WordNet model is used to weigh the class distribution similarity. It embeds class semantic information into the GNN. Using the word embedding distribution graph, the WPGN solves the problem of low classification accuracy caused by the similarity of fine-grained image features.

Second, we replace the ReLU activation function of the GNN with the FReLU [13] function. Compared with ReLU, FReLU is more suitable for processing vision tasks and can further improve classification accuracy. At the same time, according to our experiments, the Mahalanobis distance exhibits a better classification performance than Euclidean distance for FSL. Furthermore, the covariance matrix is used to eliminate the variance and dimensionality between each component. Therefore, we used Mahalanobis distance instead of Euclidean distance to calculate the distance between samples.

Finally, we combine the Efficient Channel Attention (ECA) [14] and our backbone network (ResNet-12), called ECAResNet-12, which can introduce few extra parameters, almost ignore calculations, and bring performance gain. ECAResNet-12 can better extract image feature information through one-dimensional convolution to efficiently achieve local cross-channel interaction, which extracts the dependencies between channels and avoids dimensionality reduction, and further improves the classification performance of GNN.

The remainder of this paper is organized as follows. Section 2 describes related work. Section 3 focuses on the few-shot task and introduces the framework of WPGN in detail. Section 4 presents a discussion of the results of the WPGN comparison experiments, ablation studies and practical application example, demonstrating the effectiveness of WPGN on FSL.

## 2. Related Work

Recently, there have been many high-quality works in Few-Shot Learning with novel ideas, which are different from meta-learning and metric learning. We will introduce them from four parts: graph neural network, transfer learning, semantic information, attention mechanism and application.

### 2.1. Graph Neural Network

The GNN, which is a new method for performing FSL, is a multi-layer network with a weight-sharing property. The GNN is a graph model composed of nodes and edges. Each node represents an input image, and the weight on each edge represents the relationship between different images. This relationship can consist of the distance or the similarity between the images. FSL with graph neural networks [15] transfers the distance metric from a Euclidean space to a non-Euclidean one, and its core idea is the same as that of most GNNs. It transfers the label information of labeled images to unlabeled query images. On the basis of the abovementioned GNN method, the Edge-labeling Graph Neural Network (EGNN) [16] uses edge label prediction instead of node label prediction as well as a two-dimensional edge features to explicitly express the similarities and differences between classes. Although it shows the great potential of GNN in FSL, the distribution information of the sample is ignored. Most GNN methods based on meta-learning focus on the instance-level relationship between samples, and the Distribution Propagation Graph Network (DPGN) [17] further extends this idea by explicitly simulating the distribution relationship from one instance to all other instances in a 1-vs.-*N* manner. DPGN proposes a dual-graph neural network model, which establishes the cyclic propagation process between the two graphs. The instance- and distribution-level relationships are then combined to create a better environment for few-shot classification. Unfortunately, DPGN completely ignores semantic information, which is important for fine-grained classification. Therefore, DPGN shows poor classification performance. The looking-back [18] method proposed absorbs the lower-level information in the hidden layers of the neural network using a GNN. Denoising AutoEncoders (DAE) [19] proposed a structure comprising denoising autoencoders using a graph neural network, exploited the connection between nodes to perform reconstruction, and updated the classification weight vector. However, the DAE completely ignores the semantic information of the image.

### 2.2. Transfer Learning

The transfer-learning scheme for semi-supervised few-shot learning (TransMatch) [20] uses transfer learning and weight imprinting to generate classifier weights, and it uses the MixUp method to perform semi-supervised training, demonstrating that transfer learning can achieve better results in small sample scenarios. Although the accuracy of transfer learning is lower than that of an FSL model because of its flexibility, we use this idea to extract semantic information.

### 2.3. Semantic Information

The classification method based on semantic information uses target label information as prior knowledge to assist target classification. Multiple-Semantics [21] uses a variety of semantic information to enrich the information source of small sample learning, which is closer to the situation of humans learning new things and reminds us that human beings will refer to label information when classifying. Variational AutoEncoders [22] proposed a cross and distribution aligned-variational autoencoder that combines image feature information and semantic information to construct latent features containing important multi-model information to infer the classification of unseen samples. However, methods for obtaining accurate semantic information are an important factor that limits the application of these methods. Learning Compositional Representations [23] decomposed the image representation into multiple attribute representations and improved the representation ability of the feature extraction network by adding loss-function constraints. The characteristics of manual labeling limit the applicability of the model. A classification hierarchical structure [24] uses the semantic relationship between classes to perform additional supervision on the feature extraction network and guide it to learn additional transferable feature information. This helps the KNN algorithm obtain more accurate classification results. Semantic feature augmentation in few-shot learning [25] also uses semantic information to expand the data. It encodes the feature data to map the semantic space, and it then performs classification by decoding the enhanced information. The result is better than that of data augmentation at the image level. A new hierarchical semantic embedding [26] framework effectively uses the hierarchical classification structure to guide network feature learning, encodes the correlation between different hierarchical classes, and achieves better performance on fine-grained image classification. However, this framework requires the manual annotation of datasets, which affects its practical applicability because manual annotation is tedious and time consuming. To sum up, our research focuses on acquiring accurate semantic information without manual annotation.

### 2.4. Attention and Application

Few-shot classification via adaptive attention [27] introduces a channel attention mechanism and a spatial attention mechanism to optimize feature maps. Although simpler and more effective than other models, it lacks the ability to adapt to new tasks. As there are fewer samples for learning in FSL, it means that richer features should be extracted from each image, and an attention mechanism can meet this demand. Voronoi Decomposition-based Random Region Erasing [28] generates new artificial images with randomly covered regions of irregular shape and augments the original dataset to train the neural network more efficiently. Multimodal few-shot learning for gait recognition [29] combines CNNs and RNNs using multi-modal time-series learning to map the latent embedding vector space and to address the open-set gait recognition problem. The wide application of FSL makes us realize the great research potential in this field.

Based on this research and our own work, we now introduce the WPGN and follow the dual-graph cyclic calculation method of the DPGN to construct a point graph and word embedding distribution graph that extract semantic information using the GloVe [30] model, and we eliminate the need for manual labeling, thereby achieving better classification results than extant methods.

## 3. Method

In this section, we first provide the background of our FSL task, and we then discuss our proposed WPGN in detail.

### 3.1. Problem Definition

**Figure 2:**
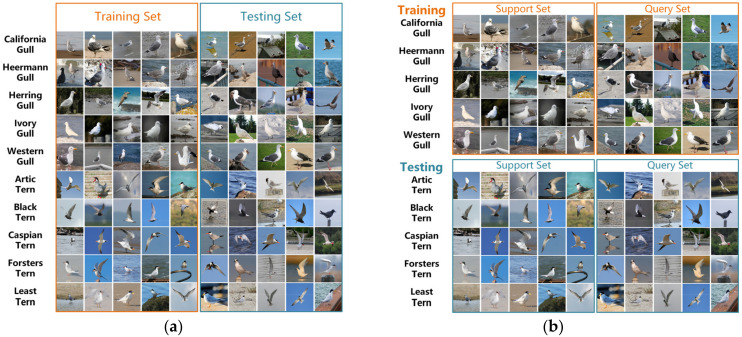
Few-shot tasks and traditional image classification tasks in CUB-200-2011 dataset: (**a**) traditional classification; (**b**) few-shot classification.

The goal of an FSL task is to train a model to perform well in cases when only few samples are given. Our few-shot task provides a support set S, a query set Q, and training image dataset D_train_. Each task contains N classes with K samples per class, which implies an N-way-K shot setting. We compare the few-shot task to the traditional image classification task in Figure 2, where Figure 2a represents the traditional image classification. We train the model on the training set in 10 classes, and then use the trained model to test the accuracy on the testing set. Conversely, Figure 2b represents a few-shot classification. There are five classes in the training, and five images of S in each class, indicating the 5-way-5 shot task category. After obtaining the trained model, the accuracy of the model is tested on the S and Q of the testing set. The testing task process is the same as the training task: a 5-way-5 shot task.

### 3.2. Feature Extraction with ECA-Net

**Figure 3:**
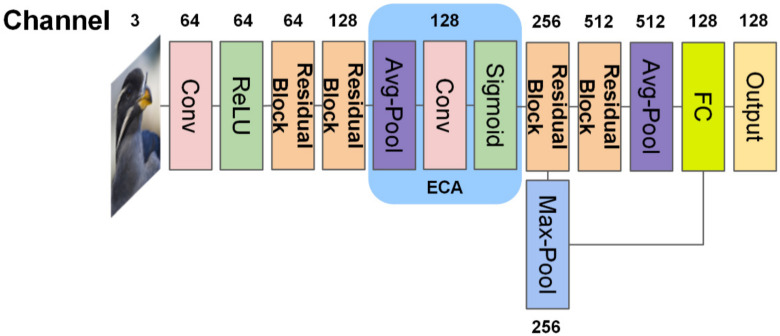
ECAResNet-12 network architecture.

The image contains a foreground and background, and the quality of feature extraction will directly affect the classification effect of the GNN. In ResNet-12, different regional features of the image are treated equally. However, in the classification task, we hope that the network can pay more attention to the foreground and ignore the background. Therefore, as shown in Figure 3, we have added a channel attention mechanism, ECA-Net, to the ResNet-12 network, to enable our backbone to ignore the background, highlight the foreground, and further improve the quality of feature extraction. In the backbone, with the increase in the number of channels, the resolution of the feature map will decrease. In the course of the channel attention learning, if either the resolution or the number of channels is too low, it will result in a decline in the image extraction quality. Because the channel attention mechanism has certain requirements on the resolution and channel number of feature map, we choose the feature map with medium channel number and resolution, that is, an ECA mechanism is added to the feature map with 128 channels.

### 3.3. Word Embedding Distribution Propagation Graph Network

As shown in Figure 1, the WPGN consists of L layers, and each layer contains a point graph and a word embedding distribution graph, which are designed based on a GNN. The ECAResnet-12 backbone network is used for feature extraction. First, an image feature is extracted in the feature extraction layer as the initialization information of the point graph. Second, according to the corresponding class label of the images, the word vectors of each class are embedded using the GloVe model to provide the initial information of the word embedding distribution graph. Third, we merge the point graph and word embedded distribution graph, update the position of the node in the point graph, and cyclically generate the point graph and word-embedded distribution graph for each layer. Finally, the distance between nodes in the point graph judges the similarity between the query set and the support set, and the class of the query set is predicted.

#### 3.3.1. Point Graph

The point graph was designed based on the GNN and generated according to the extracted image feature information. The point graph represents the position of each instance in the sample space. The nodes in the point graph are initialized as follows:

(1) $$V_{0,i}^{p}=f_{extract}(g_{i})$$

where $g_{i}$ represents each image sample instance, $f_{extract}()$ represents the backbone network for image extraction. Edge $E_{l,ij}^{p}$ in the point graph represents the image feature similarity, which can be calculated using the following formula:

(2) $$E_{l,ij}^{p}=M(V_{l,i}^{p},V_{l,j}^{p})\times E_{l-1,ij}^{p}$$

where $V_{l,i}^{p}$ and $V_{l,j}^{p}$ respectively represent nodes i and j in the point graph, and when l equals zero, $E_{l-1,ij}^{p}$ equals one. M represents the Mahalanobis distance. The calculation formula is as follows:

(3) $$M(i,j)=\frac{1}{2}{\left(i-j\right)}^{T}{\left(\tilde{Q^{\tau }}\right)}^{-1}(i-j)$$

Because FSL uses tasks as a unit of learning, we use τ for specific task and c for a class in task τ. $\tilde{Q^{\tau }}$ represents the estimated value of the covariance matrix between images in task τ. $\tilde{Q_{c}^{\tau }}$ is the estimated value of the covariance matrix between images of task τ and class c.

(4) $$\tilde{Q_{}^{\tau }}=\overset{N}{\underset{c=1}{\sum }}\frac{\tilde{Q_{c}^{\tau }}}{N}=\overset{N}{\underset{c=1}{\sum }}\frac{1}{N}(\lambda_{c}^{\tau }Q_{c}^{\tau }+(1-\lambda_{c}^{\tau })Q^{\tau })$$

where N represents the number of classes in task τ. $Q_{c}^{\tau }$ is the true value of the covariance matrix between images of class c in task τ.

(5) $$Q_{c}^{\tau }=\frac{1}{\left|K\right|}\underset{g_{i}\in K}{\sum }(f_{extract}(g_{i})-\mu_{c}){\left(f_{extract}(g_{i})-\mu_{c}\right)}^{T}$$

where $\mu_{c}$ represents the mean value of the feature embedding matrix, $f_{extract}()$, in the support set image in task τ. K represents the number of images in the support set of class c in task τ. $Q_{}^{\tau }$ is the true value of the covariance matrix between all classes of images in task τ.

(6) $$Q^{\tau }=\overset{N}{\underset{c=1}{\sum }}\frac{Q_{c}^{\tau }}{N}$$

The weight $\lambda_{c}^{\tau }$ is calculated by the following method:

(7) $$\lambda_{c}^{\tau }=\frac{K}{K+1}$$

In existing GNNs, the focus is on the use of methods for embedding information. The choice of metric is also important. Previous selection of the metric involves two unrealistic assumptions: feature dimensions are not correlated, and there is consistent covariance. Mahalanobis believes that different types of images can have different covariances, and the distribution of those images is closer to the real situation and must remain in focus. The Mahalanobis distance can deal with the problem of non-independent and identical distributions among various dimensions in high-dimensional linearly distributed data. Because the amount of data in the FSL task is small, it is important to consider the difference in image covariance of different categories. The Mahalanobis distance is the normalized distance of non-uniform distribution in Euclidean space, without considering the influence of data dimension. Furthermore, the correlation between variables is considered according to the distribution of features in the whole space. Therefore, it can better describe the similarity between data.

The Mahalanobis distance is an excellent way to solve the differences between classes; hence, the WPGN uses it to calculate the distance between them. To verify the effectiveness of Mahalanobis distance, we compare the classification results of three datasets by changing the measurement method of the similarity calculation. The experimental results are shown in Table 1.

As seen in Table 1, the Manhattan distance appears to have the lowest accuracy. The Mahalanobis distance has certain advantages compared with the Manhattan and Euclidean distances; therefore, we choose it as the best method to calculate the similarity of class in the WPGN.

#### 3.3.2. Word Embedding Distribution Graph

Similar to the point graph, the word embedding distribution graph is designed based on the GNN, which is generated by semantic information. The GloVe word embedding model is adopted to vectorize object labels in the training. WordNet [31] is used to calculate the similarity between nodes.

The GloVe model performs a vector representation of words, which makes the vectors contain as much semantic and grammatical information as possible. The GloVe model first constructs a word co-occurrence matrix based on a large corpus, and then trains word vectors with fixed dimensions, such that the distance between word vectors of different words is close to the distance between different words in the co-occurrence matrix. While reducing the dimension of data, the loss of accuracy is reduced as much as possible, and the trained word vectors are finally obtained. The word vectors of the GloVe model can be added and subtracted. For example,

(8) $$f_{g}(King)-f_{g}(Man)+f_{g}(Woman)=f_{g}(Queen)$$

where $f_{g}()$ represents the word vectors trained by the GloVe model. The fact that the word vectors can be added and subtracted allows us to create and generate it in a full sample space. This method is conducive to expanding the distance between classes in the full sample space, thus improving the classification performance. We extract the label of each class in the dataset as word vectors through the pre-trained GloVe model to use it as the initial node of the word embedding distribution graph.

The node $V_{l,i}^{w}$ in the word embedding distribution graph represents the instance of each image $g_{i}$ embedded by semantic information, and the $V_{0,i}^{w}$ initial value is as follows:

(9) $$V_{0,i}^{w}=f_{g}(label_{c})$$

where $label_{c}$ represents the label of class c.

Each edge in the word distribution graph $E_{l,ij}^{w}$ stands for the similarity between the semantic distribution features of different samples, and the calculation formula is as follows:

(10) $$E_{l,ij}^{w}=M(V_{l,i}^{w},V_{l,j}^{w})\times E_{l-1,ij}^{w}\times f_{w}(V_{l,i}^{w},V_{l,j}^{w})$$

where l equals zero, and $E_{l-1,ij}^{w}$ equals one. To prevent $E_{0,ij}^{w}$ from disappearing after many iterations of $E_{l,ij}^{w}$, the number of iteration layers is discussed in the experiment. The number of layers is set to five to avoid the disappearance of $E_{0,ij}^{w}$.

Here, $f_{w}$ represents the similarity calculated by the WordNet model, and the calculation method is as follows:

(11) $$f_{w}(x,y)=\frac{1}{abs(m_{\min}-x_{\min})+abs(m_{\min}-y_{\min})}$$

where $x_{\min}$, $y_{\min}$, and $m_{\min}$ represent the minimum depths of the word set. These three parameters are calculated as follows:

(12) $$\begin{array}{l} x_{min}=min_{depth}(x)\\ y_{min}=min_{depth}(y)\\ m_{min}=min_{depth}(m) \end{array}$$

Parameter m represents the lowest common upper word set, and the calculation formula is as follows:

(13) $$m=Low_{hy}(x,y)$$

#### 3.3.3. Loop Computation

**Figure 4:**
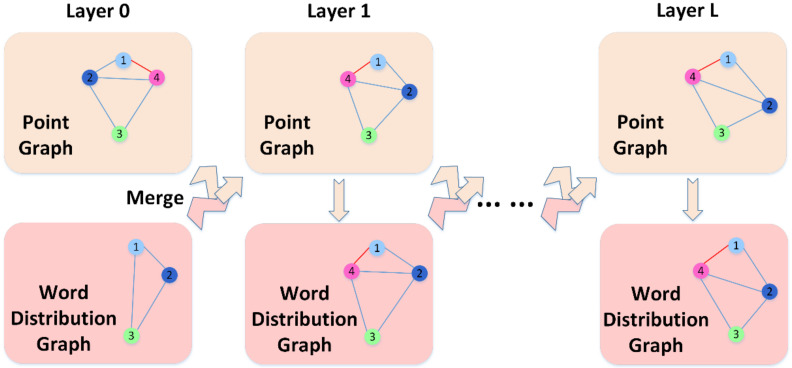
Cycle calculation process of the WPGN.

The positions between instances in the word embedding distribution graph represent the distribution of different instances in the sample space. After the WPGN is initialized, the model will perform cyclic calculations combined with word embedding to learn image features and predict the classification of the images. The cycle calculation process is shown in Figure 4.

At first, the image through feature extraction network ECAResNet-12 is used as the initial node $V_{0,i}^{p}$ of point graph 0 layer. The GloVe model is used to extract semantic information as the initial node $V_{0,i}^{w}$ of word embedding distribution graph 0 layer. Then, Mahalanobis distance is used as a measure of the distance between nodes and the edges of the point graph $E_{0,ij}^{p}$, and the word embedding distribution graph $E_{0,ij}^{w}$ is calculated. Second, $E_{0,ij}^{w}$ and $V_{0,i}^{p}$ will update the point graph through W2P and obtain point graph node $V_{1,i}^{p}$. After the calculation of $E_{1,ij}^{p}$, $E_{1,ij}^{p}$ and $V_{0,i}^{w}$, it will update the word embedding distribution graph through P2W and obtain node $V_{1,i}^{w}$. Finally, the $E_{1,ij}^{w}$ is calculated according to its nodes, where the dual-graph of layer 1 is calculated, and the calculation is repeated as described above until layer L is reached.

##### Updating the Point Graph

**Figure 5:**
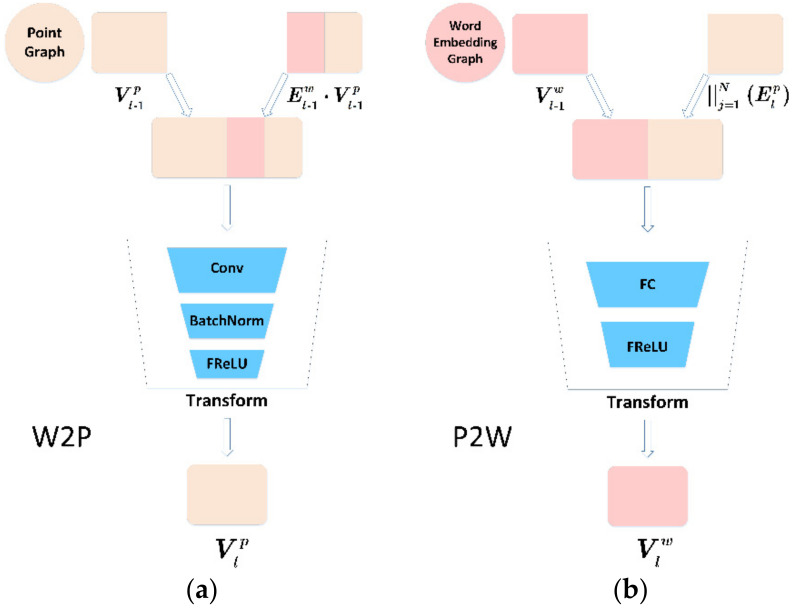
Details about W2P strategy and P2W strategy in the WPGN. (**a**) W2P; (**b**) P2W.

The process of updating the point graph is shown in Figure 5a. The point graph is adjusted in the Transform layer, which consists of a Conv layer, a BatchNorm layer, and a FReLU activation function, which transmits information in reverse. The adjustment strategy W2P is shown as follows:

(14) $$V_{l,i}^{p}=f_{FR}\left(f_{BN}\left(f_{conv2d}\left(\overset{N}{\underset{j=1}{\sum }}(E_{l-1,ij}^{w}\cdot V_{l-1,j}^{p}),V_{l-1,i}^{p}\right)\right)\right)$$

where $f_{conv2d}()$ represents the convolution operation, and $f_{BN}()$ represents the Batch Normalization operation. Then, node $V_{l,i}^{p}$ of the next layer is obtained.

##### Updating the Word Embedding Distribution Graph

The process of updating the word embedding distribution is shown in Figure 5b. The word embedding distribution graph is adjusted by the Transform layer, which includes the full connection layer and the FReLU activation function, which provides fusion transfer adjustments. We propagate the query set images without label information from the point graph to the word embedding distribution graph. The node adjustment method P2W is as follows:

(15) $$V_{l,i}^{w}=f_{FR}(f_{FC}(|\;{|}_{j=1}^{N}(E_{l,ij}^{p}),V_{l-1,i}^{w}))$$

where | | is the concatenation operator, which aggregates scalar $E_{l,ij}^{p}$ into vectors.

##### FReLU Activation Function

FReLU is a simple and effective activation function suitable for processing visual tasks. It improves the ReLU by adding negligible spatial conditions overhead. FReLU is more suitable for the GNN than ReLU. We use FReLU to combine features in the Transform layer of W2P and P2W in the same way that WPGN obtains the point graph and word embedding distribution graph of the next layer. The FReLU is calculated as follows:

(16) $$f_{FR}(x)=Max(x,T(x))$$

where T(x) represents a simple and efficient spatial context feature extractor. T(x) is defined as follows:

(17) $$T(x)=f_{BN}(f_{conv2d}(x))$$

To verify the effectiveness of FReLU in the WPGN, we compare the classification results using different activation functions on the CUB-200-2011 dataset. The experimental results are shown in Table 2.

Here, the activation function has a non-negligible impact on the WPGN model. Compared with the LeakyReLU, FReLU used in this study has a certain degree of accuracy improvement. From ReLU to the latest FReLU, the accuracy of the WPGN has increased by 1.83%.

#### 3.3.4. Loss Function

We use the Softmax function as the classification function, combining point graph loss and word embedding distribution graph loss as the loss value of the WPGN. The prediction process of each node is as follows:

(18) $$P\left(y_{i}|x_{i}\right)=Softmax\left(\overset{K}{\underset{j=1}{\sum }}E_{l,ij}^{p}\cdot \mathrm{one-hot}(y_{j})\right)$$

where $P(y_{i}|x_{i})$ is the most probable class, given sample $x_{i}$ that belongs to the point graph, and $y_{i}$ is the label of the j th sample in the support set.

The calculation steps of the loss function are as follows.

1. Calculate the point graph loss:
  (19) $$L_{l}^{p}=L_{CE}(P(y_{i}|x_{i}),y_{i})$$
  where $L_{l}^{p}$ represents the loss of the L-layer point graph, and $L_{CE}$ is the cross-entropy loss function.
2. Calculate the word embedding distribution graph loss:
  (20) $$P_{w}\left(y_{i}|x_{i}\right)=\mathrm{Softmax}\left(\overset{K}{\underset{j=1}{\sum }}E_{l,ij}^{w}\cdot \mathrm{one-hot}(y_{j})\right)$$
  where $P_{w}(y_{i}|x_{i})$ is the most probable class, given sample $x_{i}$ that belongs to the word embedding distribution graph.
  (21) $$L_{l}^{w}=L_{CE}(P_{w}(y_{i}|x_{i}),y_{i})$$
3. Calculate the model loss.

To balance the two losses, we introduce weight λ and calculate the total loss as follows:

(22) $$L_{loss}=\lambda L_{l}^{p}+(1-\lambda )L_{l}^{w}$$

The classification accuracy is shown in the Table 3 as *λ* takes different values.

It can be seen from Table 3, with the increase in *λ* value, the classification accuracy gradually improves. When *λ* is 0.9, the highest accuracy rate can be obtained, and when it is greater than 0.9, the classification accuracy begins to decrease. The WPGN obtains the minimum loss value when *λ* is 0.9; thus, we set *λ* to 0.9.

## 4. Experiment

### 4.1. Experimental Environment and Datasets

The experimental environment of this paper is shown in Table 4.

We selected three types of standard datasets in FSL: MiniImageNet [9], CUB-200-2011 [32] and CIFAR-FS [4]. The details of the images, classes, training/validation/test set divisions and the image resolutions of each dataset are shown in Table 5.

**Figure 6:**
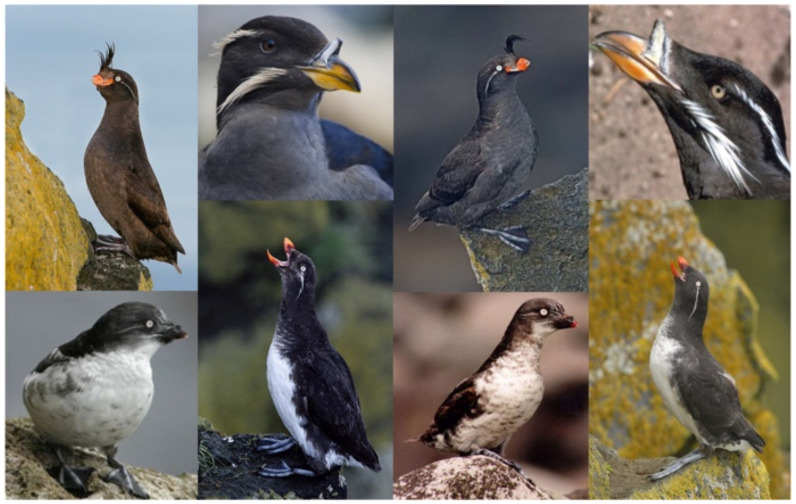
Four different classes of birds in CUB-200-2011.

As illustrated in Figure 6, the image features of the four different classes of birds in the CUB-200-2011 dataset are similar and more difficult to distinguish.

### 4.2. Experimental Settings

**Figure 7:**
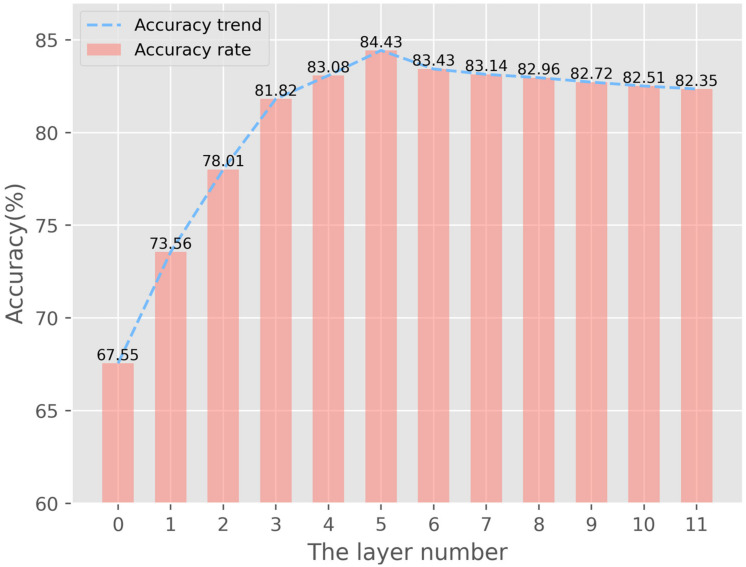
Effects of different layer numbers on classification accuracy.

The WPGN uses cyclic computation to construct the network structure, including the point graph and word embedding distribution graph. Mutual updating between the dual-graph is the biggest feature of the WPGN. Therefore, the total number of layers of the WPGN affects the final classification results. To find the layer number that best fits the network structure, we trained the WPGN on the CUB-200-2011 dataset by changing the layer number to obtain the classification accuracy of each training model. The experimental results are shown in Figure 7.

Here, we can see that the abscissa represents the number of layers, zero represents no cyclic calculation, and one represents one cycle calculation. When the layer number increases from zero to five, the classification accuracy increases by nearly 17%. However, the growth of classification accuracy tends to be flat and slightly oscillates when the layer number is greater than five. Therefore, we chose five as the final layer number of the WPGN.

To more intuitively show the impact of different layer numbers on the WPGN’s classification accuracy, the labeled class [1,2,3,4,5] was selected for the experiment, and a heat map was used to show the change of classification accuracy with the increase in layer numbers.

**Figure 8:**
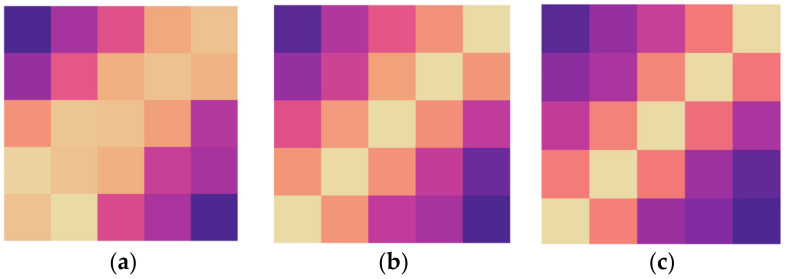

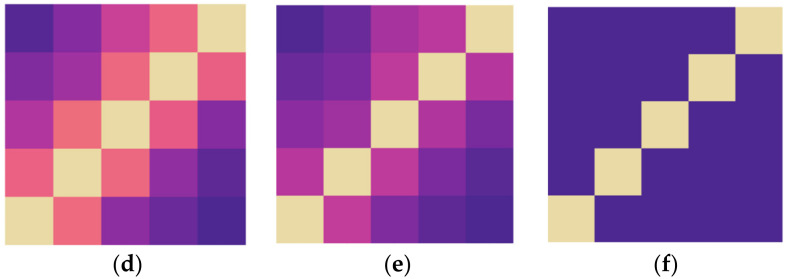
Impact of different layer numbers in the WPGN on classification accuracy: (**a**) layer 0; (**b**) layer 1; (**c**) layer 2; (**d**) layer 3; (**e**) layer 5; (**f**) ground truth.

The brighter parts indicate high confidence. Figure 8a did not use a cycle for calculation; hence, the classification accuracy was low, resulting in fuzzy predictions and a greater possibility of predicting the wrong label. Figure 8e has five layers, and with the exception of the ground-truth location, the other parts have darker colors, meaning that the probability of an accurate prediction is much higher than that of the prediction error.

The parameter settings obtained in the WPGN are shown in Table 6 and listed by experiment.

### 4.3. Evaluation

In this paper, classification accuracy was used to evaluate the performance of the model. The higher the accuracy, the better the performance of the model. We randomly selected n = 10,000 tasks, and we published the mean accuracy and the 95% confidence interval. The calculation formula of accuracy is as follows:

(23) $$\overset{n}{\underset{i=1}{\sum }}\frac{Acc_{i}}{n}$$

### 4.4. Experimental Results

For this study, we used ConvNet, RestNet-12 and ECAResNet-12 as the backbone network of features traction with three tasks: 5-way-1 shot/2 shot/5 shot. The experimental results are shown in Table 7 on the CUB-200-2011 dataset.

As can be seen from Table 7, the classification accuracy of the WPGN under three backbone networks and three tasks is higher than the other methods. When the feature extraction backbone network is ECAResNet-12 and the tasks are 5-way-1 shot, 5-way-2 shot, and 5-way-5 shot, the accuracy of the WPGN is improved by nearly 9.0%, 4.5%, and 4.1%, respectively, compared with the DPGN. The accuracy of the WPGN under the 5-way-2 shot task is approximately 2% higher than that of the DPGN under 5-way-5 shot. The experimental results prove and demonstrate that our WPGN is robust in fine-grained classification.

**Figure 9:**
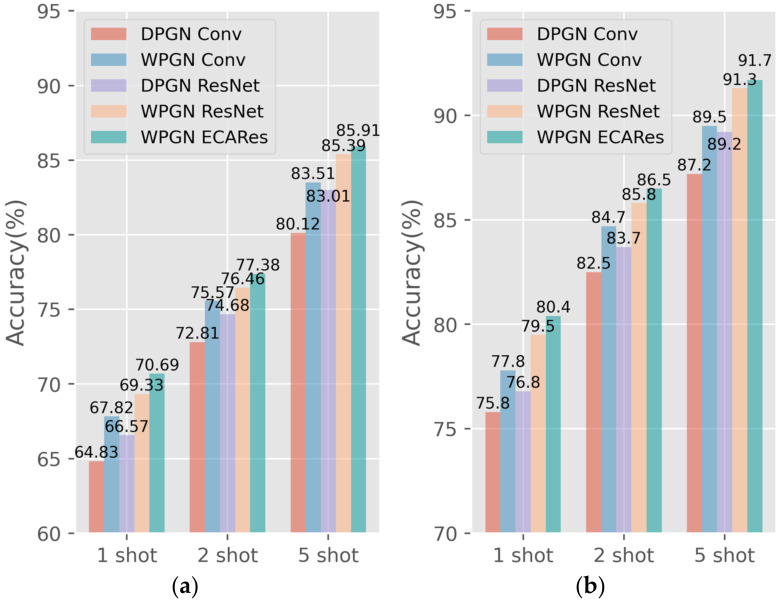
Experimental results on the MiniImagenet and CIFAR-FS. (**a**) MiniImagenet; (**b**) CIFAR-FS.

The experimental results are shown in Figure 9 on the MiniImagenet and CIFAR-FS datasets.

Here, the DPGN Conv represents the feature extraction backbone network as ConvNet on the DPGN, the WPGN ResNet represents the feature extraction backbone network as ResNet-12 on the WPGN, whereas the WPGN ECARes represents the feature extraction backbone network as ECAResNet-12 on the WPGN. From Figure 9, we can see that on the MiniImagenet dataset and the CIFAR-FS dataset, the classification accuracy of the WPGN is higher than that of the DPGN on the three tasks. Moreover, when the feature extraction backbone network adopted ECAResNet-12, its classification effect was better than that of ConvNet and ResNet-12. The experiment demonstrated that the WPGN performs better in a dataset having fewer obfuscating features. The accuracy of the CIFAR-FS dataset was lower than that of the MiniImagenet dataset because its background had a much smaller impact on the classification accuracy.

Moreover, compared with the DPGN, our model had less computational overhead while improving accuracy. This is because FSL is trained in task units. For each task, the first layer of the DPGN distribution graph requires a large number of calculations to initialize, whereas the first layer of the word distribution graph associated with the WPGN only needs to obtain the word vectors of the corresponding category. Thus, initialization is completed much faster. The time used for the same number of steps of WPGN and DPGN training is shown in Table 8. For the same number of rounds of training, WPGN requires far fewer calculations than DPGN.

**Figure 10:**
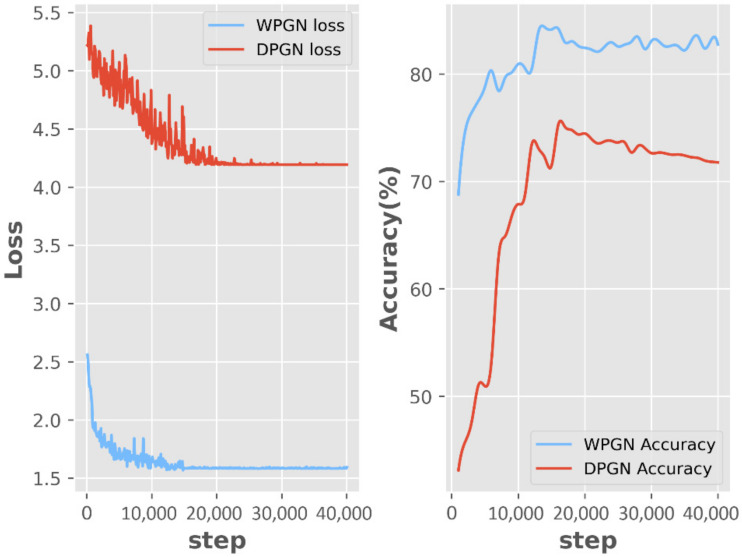
Training loss and test accuracy comparison.

In addition, compared with the number of training rounds, as shown in the left side of Figure 10, the loss convergence speed of WPGN is significantly faster than that of DPGN, which shows that the WPGN is better in total training time. We found that WPGN converged in 12,000 rounds. Hence, we reduced the learning rate for further optimization. DPGN requires at least 15,000 rounds to converge such that the learning rate can be reduced. We tried to reduce the learning rate for DPGN at 12,000 rounds, but experimental results show that the accuracy of DPGN was reduced by ~2%. The right side of Figure 10 shows that, compared with DPGN, WPGN converges faster and significantly improves test accuracy. At the same time, after several rounds of training, the performance of WPGN will not obviously decline, which proves that the model is more robust and less prone to overfitting.

Because the WPGN model is better than the DPGN in terms of calculation overhead and accuracy, it demonstrates that our research content has a good prospect for real-world applications.

### 4.5. Ablation Studies

In order to verify the validity of each component in WPGN, we will add word embedding, Mahalanobis distance, FReLU and ECA to the baseline model one by one. In the baseline model, the distance measurement method is Euclidean distance, and the activation function is the Leakey ReLU function. The ablation experiment can fully prove the effectiveness of the components. The results of the ablation experiment on the CUB-200-2011 and CIFAR-FS datasets under 5-way-1 shot tasks are shown in Table 9.

As observed from the results in the table, after the word embedding distribution graph was added to the WPGN, the classification accuracy of the two datasets increased by 7.23% and 2.1%. Using Mahalanobis distance in the similarity calculation method, the classification accuracy increased by ~0.4%. The activation FReLU function also improved the classification accuracy of the model, and although it is not as great as the first two innovations, it contributes to the improvement of model accuracy. Finally, by integrating the ECA attention module into ResNet-12, the accuracy of our model has increased by 1.2%. From the experimental results, it can be seen that for these two datasets, the four innovations described in this paper improved the classification accuracy of the model.

### 4.6. Practical Application Example

**Figure 11:**
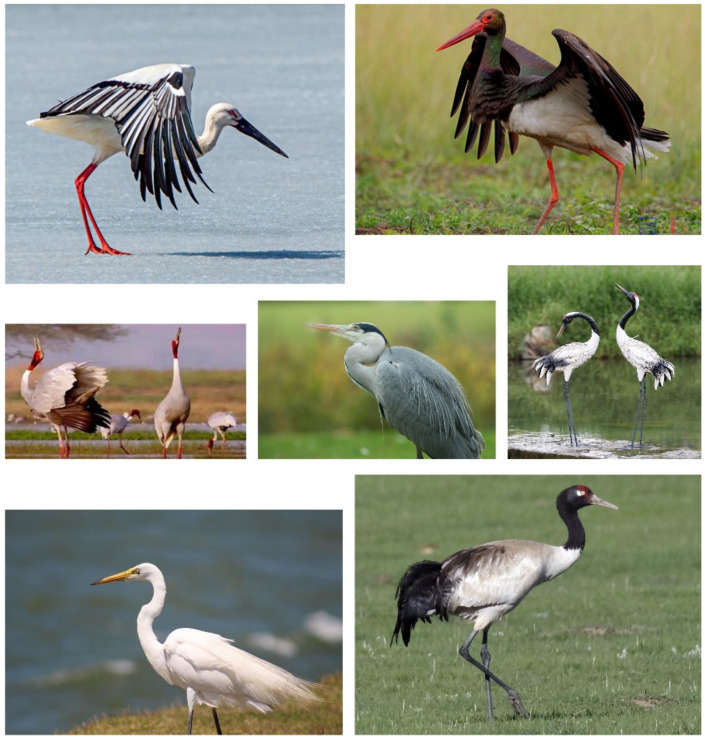
Practical application example of rare bird classification.

In order to prove the great potential of WPGN in practical application, we added an example to apply the trained WPGN to the classification of specific rare birds. In this case, seven species of rare birds in bird habitats were selected, as shown in Figure 11, of which two belonged to storks as shown in the upper part and five belong to cranes at the bottom. We can see that the similarity between these birds is high, although these birds belong to different categories. It is difficult for ordinary people to distinguish these seven species of birds if you are not a professional ornithologist. Generally speaking, compared with common image classification problems, fine-grained classification faces the images with more similar appearance characteristics. In addition, there are interference factors such as posture, illumination, viewing angle, occlusion and background in the collection, which lead to the characteristics of small differences between classes and large differences with classes. By using category labels, WPGN can first increase the distance between storks and cranes, and the distance between storks and cranes from the word vectors will be greater than the distance between subcategories. Second, in the subcategories of cranes or storks, word vectors can also divide well according to the category labels. Finally, image information is embedded into GNN to classify birds with the aid of semantic information.

The example contains 350 images of seven species of birds, and we use WPGN trained on the CUB-200-2011 dataset to test this example with 7-way-1 shot task. In this example, the accuracy of WPGN on the 7-way-1 shot task is 82.45%, while our baseline model is 72.14%. Importantly, semantic information can be obtained without manual tagging. This example illustrates the huge potential of WPGN in practical application.

## 5. Conclusions

In this paper, we proposed the WPGN. To the best of our knowledge, this is the first attempt at combining semantic information with the GNN in FSL. Several experiments showed that our method achieves state-of-the-art results in fine-grained FSL. Compared with the baseline, the 5-way-1 shot task was improved by nearly 9%. At the same time, we can see that WPGN has a greater accuracy improvement on the CUB-200-2011 dataset than the other two datasets. The CUB-200-2011 dataset is a fine-grained dataset containing 200 categories of birds. Because of the great similarity between the classes, the fusion of semantic information and visual information can play a better role in this application scenario. In the use of semantic information, we used the GloVe model to extract word vectors, which greatly enhanced the practicability of this method. The effect of WPGN in the classification of rare birds shows that the method is flexible and feasible in practical applications. However, there are some limitations to our model. The WPGN fails to integrate semantic information with the GNN to the maximum extent and must therefore be improved in this regard. In future works, we will explore further improved methods to embed semantic information in fine-grained FSL to improve the influence of semantic information on classification accuracy. The uncertainty of a graph neural network in terms of layers will also be the focus of our future work.

## Figures and Tables

**Table 1:** Different measurement methods.

	Distance	Manhattan	Euclidean	Mahalanobis
Dataset				
CUB-200-2011		83.51	83.81	84.34
MiniImageNet		69.92	70.34	70.69
CIFAR-FS		79.6	79.9	80.4

**Table 2:** Effectiveness of introducing FReLU into the WPGN.

Function	ReLU	PReLU	Swish	LeakyReLU	FReLU
Acc	82.51	82.92	83.55	83.95	84.34

**Table 3:** *λ*’s influence on classification accuracy.

*λ*	0.1	0.2	0.3	0.4	0.5
Acc	81.86	82.32	82.59	82.85	83.15
*λ*	0.6	0.7	0.8	0.9	1.0
Acc	83.32	83.51	83.82	84.34	83.88

**Table 4:** Hardware and software environments.

GPU	Python	Torch	CUDA	Torchvision	Torchnet
1080Ti	3.5.2	1.1	10.1	0.3.0	0.0.4

**Table 5:** Details for few-shot dataset benchmarks.

Dataset	Images	Classes	Train/Val/Test	Resolution
MiniImageNet	60 k	100	64/16/20 [9]	84 × 84
CUB-200	11.7 k	200	100/50/50 [33]	84 × 84
CIFAR-FS	60 k	100	64/16/20 [4]	32 × 32

**Table 6:** Hyperparameter settings.

Parameter	Value
Adam learning rate	10^−3^
Decay learning rate	10^−1^
Decay iterations	12,000
Weight decay	10^−5^
Layer number	5

**Table 7:** Few-shot classification accuracies on CUB-200-2011.

Model	Backbone	1 Shot	2 Shot	5 Shot
MAML	ConvNet	55.92 ± 0.87	/	72.09 ± 0.76
MatchingNet	ConvNet	61.16 ± 0.95	/	72.86 ± 0.69
RelationNet	ConvNet	62.45 ± 0.89	/	76.11 ± 0.66
CloserLook	ConvNet	60.53 ± 0.87	/	79.34 ± 0.69
DPGN (Batch size: 30)	ConvNet	75.52 ± 0.59	85.65 ± 0.52	89.31 ± 0.51
DPGN (Batch size: 40)	ConvNet	76.05 ± 0.51	/	89.08 ± 0.38
WPGN (Batch size: 30)	ConvNet	81.25 ± 0.46	88.62 ± 0.38	92.65 ± 0.42
FEAT	ResNet-12	68.87 ± 0.22	/	82.90 ± 0.15
DPGN (Batch size: 30)	ResNet-12	75.31 ± 0.31	87.72 ± 0.41	90.26 ± 0.30
DPGN (Batch size: 40)	ResNet-12	75.71 ± 0.47	/	91.48 ± 0.33
WPGN (Batch size: 30)	ResNet-12	83.05 ± 0.45	91.31 ± 0.34	93.91 ± 0.33
WPGN (Batch size: 30)	ECARes-12	84.34 ± 0.66	92.28 ± 0.41	94.41 ± 0.28

**Table 8:** Training time comparison.

	WPGN	DPGN
Steps	40,000	40,000
Time (minutes)	416	724

**Table 9:** Ablation experiment on CUB-200-2011 and CIFAR-FS.

Datasets	Word Embedding	Mahalanobis Distance	FReLU	ECA Attention	Accuracy
	×	×	×	×	75.31
	√	×	×	×	82.54
CUB-200-2011	√	√	×	×	82.91
	√	√	√	×	83.10
	√	√	√	√	84.34
	×	×	×	×	76.8
	√	×	×	×	78.9
CIFAR-FS	√	√	×	×	79.2
	√	√	√	×	79.5
	√	√	√	√	80.4

## Data Availability

Publicly available datasets were analyzed in this study. This data can be found here: CUB-200-2011: https://resolver.caltech.edu/CaltechAUTHORS:20111026-120541847, MiniImagenet: https://www.image-net.org/, CIFAR-FS: DOI: 10.1109/IROS45743.2020.9341282.
